# The Probiotics *Lacticaseibacillus paracasei*, *Lacticaseibacillus rhamnosus*, and *Limosilactobacillus fermentum* Enhance Spermatozoa Motility Through Mitochondrial Function-Related Factors

**DOI:** 10.3390/ijms252313220

**Published:** 2024-12-09

**Authors:** Eun Hye Lee, Yu Jin Kim, Il Seon Jung, Dae Keun Kim, Jae Ho Lee

**Affiliations:** 1 Department of Biomedical Science, College of Life Science, CHA University, Pocheon 11160, Gyeonggi-do, Republic of Korea; whitenum@naver.com; 2 CHA Fertility Center, Seoul Station, Hangang-daero, Jung-gu, Seoul 04637, Republic of Korea; yj_kim@chamc.co.kr; 3 Institute for Commercialization of Commensals Microbiota (ICCM), Biostream Co., Ltd., 46 Cheongmyeongsan-ro, Giheung-gu, Yongin-si 17098, Gyeonggi-do, Republic of Korea; isjung@biostream.co.kr; 4 Department of Urology, CHA Fertility Center Seoul Station, CHA University School of Medicine, Seoul 04637, Republic of Korea

**Keywords:** spermatozoa, probiotics, motility, viability, mitochondrial activity

## Abstract

Idiopathic male infertility is characterized by increased mortality or reduced motility and vitality of sperm. There are several reports on probiotics in the male reproductive tract, but the effects of these probiotics on sperm motility remain to be elucidated. In this study, we investigated the impact and mechanism of probiotics on the vitality and motility of mouse sperm. We collected mature sperm from the caudal vas deferens of mice and prepared three probiotics donated by HEM Pharma Inc.: *Lacticaseibacillus rhamnosus*, *Limosilactobacillus fermentum*, and *Lacticaseibacillus paracasei*. We analyzed the vitality and motility of sperm according to the concentration and duration of probiotic treatment. The probiotics increased the motility and vitality of sperm. Specifically, they enhanced sperm motility by 30–40% compared with untreated sperms. The probiotics enhanced mitochondrial activity in sperm through specific factors like AMPK and SIRT1. All three probiotics enhanced the activities of mitochondrial function-related proteins in sperm. In conclusion, we found that the probiotics improved the vitality and motility of mouse sperm and increased mitochondrial function in mature sperm. These findings suggest that probiotics can be utilized to enhance sperm motility and treat male infertility.

## 1. Introduction

Male fertility is evaluated by semen analysis based on the guidelines of the World Health Organization [1]. Sperm motility is one of the most important factors for sperm to reach the female reproductive tract and fertilize an oocyte. The energy required for sperm motility is provided by mitochondria, which are mainly distributed in the mid-neck region. Energy generated by mitochondria is used to phosphorylate flagellar proteins [2,3,4]. This initiates or maintains sperm motility, which is essential for normal fertilization.

Probiotics have been found in most human organs, including male reproductive organs [5]. Specific probiotics have been found in seminal plasma [6]. A few studies have reported that specific probiotics are associated with male infertility and semen quality [7]. However, the effect of probiotics in seminal plasma on sperm motility and the underlying mechanism have not been clearly elucidated. There is strong evidence that probiotics may affect factors that increase the ability of sperm to fertilize oocytes, such as sperm motility [5,6,8,9]. For example, certain gut bacteria modulate expression of genes involved in testosterone synthesis. However, further research is needed to fully understand the relationship between the gut microbiota and sperm motility.

Sperm motility is associated with production of adenosine triphosphate (ATP) by mitochondria, and ATP supports serine and tyrosine phosphorylation of sperm flagellar proteins [3,4]. Tyrosine phosphorylation of proteins in sperm tails leads to hyperactive sperm motility, which is required for oocyte fertilization [2]. However, energy loss occurs upon perturbation of mitochondrial function [10]. Interestingly, a neuroscience group reported that probiotics improve mitochondrial function and bioenergetics [11]. Therefore, probiotics could potentially increase sperm motility by modulating mitochondrial activity; however, the mechanism will need to be addressed before probiotics can be considered for use in clinical applications. 

In this study, we investigated the relationship between probiotics and sperm motility, including the functional activation mechanism of probiotics on mitochondria.

## 2. Results

### 2.1. Optimal Probiotic Treatment for Sperm Survival

**Figure 1:**
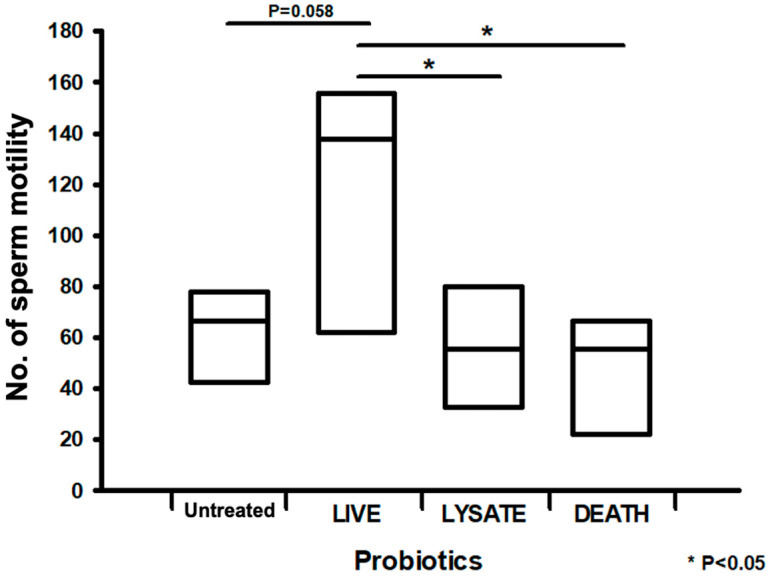
Effects of treatment with live, lysed, and dead *L. rhamnosus* on sperm motility. The bar graph presents the motility of spermatozoa treated with live, lysed, and heat-killed probiotics. All data are means ± SEM of triplicate values. * *p* < 0.05 versus untreated.

We first investigated the optimal conditions for determining the effects of live, lysed, and dead *L. rhamnosus* on sperm motility. Figure 1 shows that live probiotic treatment resulted in a maximum improvement in sperm motility of 70% compared with the untreated group. Conversely, treatment with lysed or dead probiotics did not improve sperm motility (Figure 1). Therefore, we used live other probiotics in subsequent experiments.

### 2.2. Probiotics Improve the Motility and Viability of Spermatozoa

**Figure 2:**
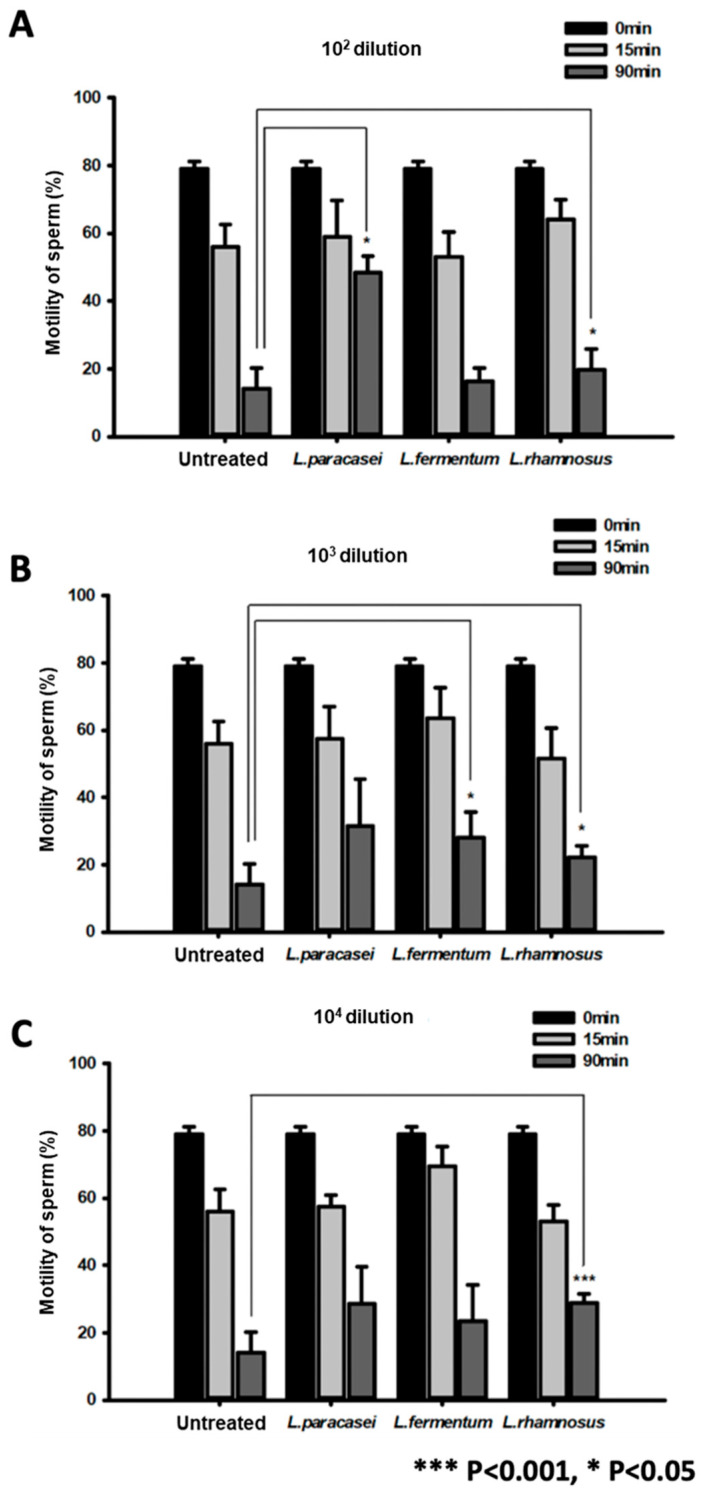
Optimal treatment of spermatozoa with probiotics. The bar graphs present the viability of spermatozoa treated with 10^2^ (**A**), 10^3^ (**B**), and 10^4^ (**C**) dilutions of probiotics for 0, 15, and 90 min. * *p* < 0.05 and *** *p* < 0.001, versus untreated.

**Figure 3:**
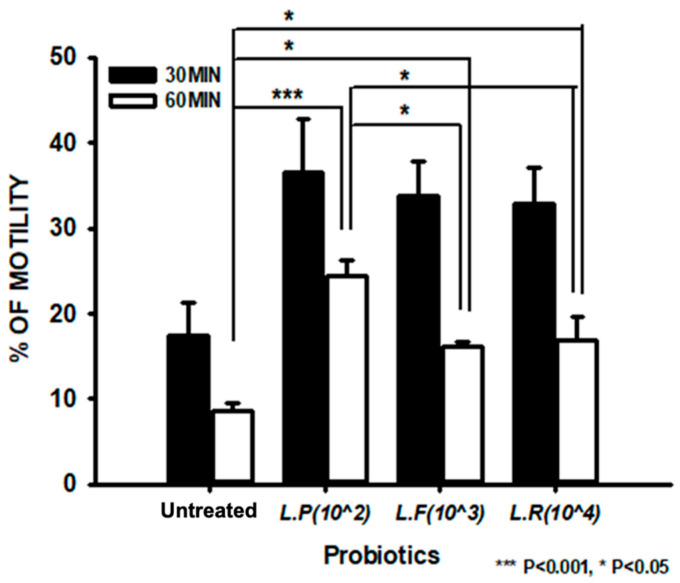
Sperm motility analysis upon treatment with probiotics. The bar graph presents the motility of spermatozoa following treatment with probiotics for 30 and 60 min. All data are means ± SEM of triplicate values. * *p* < 0.05 and *** *p* < 0.001 versus untreated.

We studied the optimal concentration range for probiotic treatment to improve sperm motility. The effects did not significantly differ when sperm were treated with probiotics at concentrations from 10^4^ to 10^2^ dilution for 0, 15, and 90 min (Figure 2A–C). The influence of probiotics on sperm cell motility was investigated. Treatment with probiotics improved the motility of sperm cells compared with the untreated group (reported as percentages) (Figure 3).

**Figure 4:**
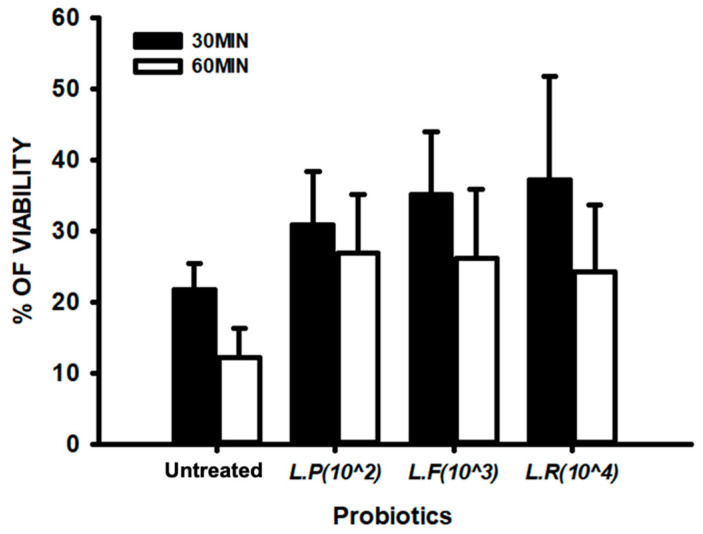
Sperm viability analysis upon treatment with probiotics. The bar graph presents the viability of spermatozoa following treatment with probiotics for 30 and 60 min. All data are means ± SEM of triplicate values.

We further quantitatively evaluated the effects of probiotics on sperm cell motility. Treatment with each probiotic enhanced sperm cell motility by two times relative to the untreated group (Table S1). Probiotic treatment did not significantly increase sperm cell viability relative to the untreated group (Figure 4, Table S2).

### 2.3. Mechanism by Which Probiotics Enhance Sperm Motility

**Figure 5:**
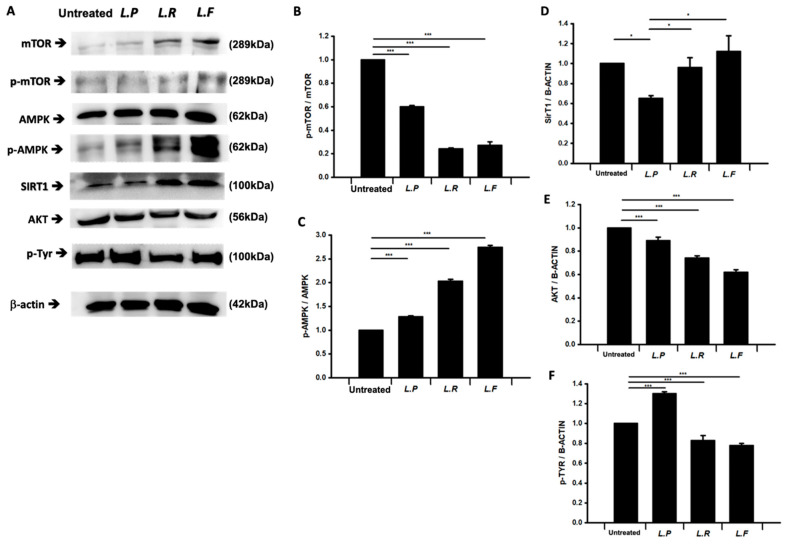
Quantification of mitochondrial function-related factors by western blotting. (**A**) Immunoblots of mTOR, phospho-mTOR, AMPK, phospho-AMPK, AKT, SIRT1, phospho-Tyr, and β-actin following treatment of spermatozoa with probiotics. (**B**–**F**) The bar graphs present the normalized band intensities of phospho-mTOR (**B**), p-AMPK (**C**), SIRT1 (**D**), AKT (**E**), and phospho-tyrosine (**F**). All data are means ± SEM of triplicate measurements. * *p* < 0.5, *** *p* < 0.001 versus untreated.

Probiotics significantly enhanced the motility of sperm cells. Therefore, we analyzed factors related to mitochondrial functional activity such as mTOR, AMPK, AKT, and SIRT1 in probiotic-treated sperm cells by western blotting. In general, the motility of sperm cells is tightly related to the functional activities of mitochondria. Interestingly, treatment of sperm cells with probiotics significantly altered the phosphorylation ratios of AMPK and mTOR and the level of SIRT1 relative to the untreated group (Figure 5A–D).

While probiotic treatment increased the total level of mTOR, it decreased the phosphorylation ratio of mTOR compared with the untreated group (Figure 5A,B). The level of SIRT1 was significantly increased in *Lacticaseibacillus rhamnosus*- and *Limosilactobacillus fermentum*-treated sperm compared with the untreated group (Figure 5A,D). The level of AKT was decreased in probiotic-treated sperm (Figure 5A,E). To investigate whether increased phosphorylation of tyrosine in sperm tails induced by probiotics affects sperm activation, we performed western blot analysis of tyrosine phosphorylation using an anti-p-Tyr antibody (Figure 5A,F). The level of tyrosine phosphorylation was significantly increased by 10–20% in *Lacticaseibacillus paracasei*-treated spermatozoa compared with the untreated group, but was not increased in *L. rhamnosus*- and *L. fermentum*-treated spermatozoa.

## 3. Discussion

In this study, we found that optimal probiotic treatment enhanced sperm motility. *L. rhamnosus* and *L. paracasei* promoted sperm motility, ranging from low-movement patterns to rapid movements, including hyperactivation and straight movements. Modulation of sperm motility by probiotics requires ATP produced by mitochondria and mechanical regulation of the fibrous sheath for the strong planar beating of flagella. Probiotics increased AMPK phosphorylation and, in some cases, tyrosine phosphorylation in sperm.

**Figure 6:**
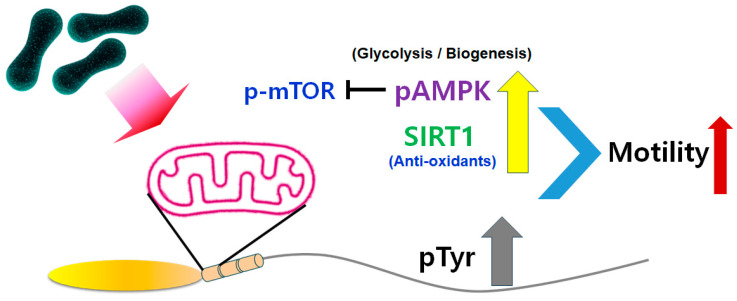
Signaling pathway underlying the effect of probiotics on sperm motility. Probiotics increase phosphorylation of AMPK and the level of SIRT1, and thereby reduce phosphorylation of mTOR. Finally, probiotics enhance phosphorylation of tyrosine to increase sperm motility.

We elucidated the mechanism by which probiotics modulate sperm motility (Figure 6). The effects of probiotics and the underlying mechanisms remain unclear in mammals. In particular, it is debated which energy production system is involved in mitochondrial aerobic respiration. We found that probiotics activated several mitochondrial function-related proteins. Normal sperm motility requires energy and the activation of flagellar proteins such as dyneins in their tails, which is mediated by phosphorylation with kinases such as protein kinase A. ATP produced by mitochondrial aerobic respiration is a major energy source for sperm motility. ATP produced by mitochondria is converted to cyclic adenosine monophosphate, which induces activation of protein kinase A [5]. We hypothesize that probiotics induce the release of anti-reactive oxygen species factors to protect against mitochondrial dysfunction in sperm and enhance mitochondrial aerobic respiration to maintain sperm motility and that the large amount of ATP produced by mitochondria promotes tyrosine phosphorylation to enhance sperm motility. *L. paracasei*-treated sperm had a high level of tyrosine phosphorylation. Sperm motility is enhanced by tyrosine phosphorylation. Tyrosine phosphorylation of sperm flagellar proteins is also associated with hyperactivated sperm motility.

Asthenozoospermia is characterized by lowly motile or immotile sperm and prevents natural fertilization [12], but its cause remains to be elucidated. This has prevented the development of clinical treatments, such as intracytoplasmic sperm injection [13]. Sperm motility in asthenozoospermia correlates with the amount of ATP generated by electron transport between proteins in the mitochondrial membrane [14]. Electron supply is important for ATP synthesis, carried out by several enzymes (nicotinamide adenine dinucleotide and flavin adenine dinucleotide) involved in electron transport in the mitochondrial membrane [10]. Mitochondrial dysfunction reduces aerobic energy production and leads to changes at the tissue level depending on metabolic demands. Spermatozoa with reduced motility have mutations in mitochondrial DNA and deletions of genes encoding complexes I and II of the respiratory chain in the mitochondrial membrane [15,16]. Mitochondrial dysfunction is related to poor sperm motility in patients with asthenozoospermia due to reductions in energy production [15]. Probiotics increase energy production by sperm mitochondria and thus are a potential treatment for low sperm motility.

Probiotic-treated sperm showed significant increases in AMPK phosphorylation and SIRT1 activation (Figure 6). SIRT1 in sperm is a major factor affecting the bioenergy metabolism in mitochondria that is required for sperm motility [17,18,19,20]. The activated, phosphorylated form of AMPK decreases mTOR phosphorylation to maintain mitochondrial activity [21]. The phosphorylation of AMPK is involved in key physiological functions of sperm, such as increasing sperm motility through bioenergy metabolism in mitochondria. Methods have been developed in the assisted reproductive field that stimulate or modulate AMPK activity in sperm. Therefore, AMPK activators such as *L. rhamnosus* and *L. paracasei* could be used to increase sperm motility. Additionally, SIRT1 is reported to have an antioxidant effect on the deacetylation and ADP-ribosylation of numerous substrates in a nicotinamide adenine dinucleotide (NAD+ (cofactor))-dependent manner in sperm mitochondria [17]. SIRT1 has a key role in normal sperm motility because Sirt1-knockout mice show a range of phenotypic characteristics, including small testes, decreased sperm counts, reduced sperm motility, and abnormal spermatozoa. Taken together, the results of this study show that live probiotics and their metabolites modulated mitochondrial bioenergy generation in sperm. Further study will be needed to identify the specific metabolite factors in probiotics that enhance sperm motility.

Probiotics improved sperm motility by enhancing mitochondrial energy production and may recover sperm motility in patients with asthenozoospermia. Additional studies are needed to identify the factors involved in recovery of metabolic and tail defects in immotile sperm. Some probiotics may require normal sperm with functional mitochondria and tails to enhance sperm motility.

## 4. Materials and Methods

### 4.1. Propagation of Probiotics

*Lacticaseibacillus rhamnosus* HEM0042, *Limosilactobacillus fermentum* HEM0117, and *Lacticaseibacillus paracasei* HEM0020 were donated by HEM Pharma Inc. (Youngin, Gyeonggi-do, Republic of Korea). For laboratory-scale propagation of these three strains, MRS broth (Difco^TM^ Lactobacilli MRS Broth, BD, Detroit, MI, USA) was used. All strains propagated by MRS broth culture (37 °C for 48 h) were harvested by centrifugation and then preserved in 25% glycerol at −70 °C prior to use. For in vitro/vivo tests, the strains were prepared through two propagation steps (5.0%, *v*/*v* inoculum size) in industrial media containing food-grade industrial ingredients. All strains were of human origin and prepared in anoxic conditions at 37 °C for 48 h.

### 4.2. Cultivation of Probiotics

All samples were prepared through a series of cultures using industrial media of food grade after activation. MRS broth was used for static activation of all strains in anoxic conditions (37 °C for 12 h) immediately after they were removed from the freezer (−70 °C). All strains activated through growth in MRS broth were inoculated (5.0% (*v*/*v*)) into the seed culture. The seed cultures were conducted with MRS broth in anoxic conditions at 37 °C for 10–12 h depending on the growth rate without addition of alkaline solution for pH control.

The growth media for all strains were prepared with food-grade ingredients. They contained the following (% *w*/*v*): 5.0% glucose, 1.0% soy peptone, 1.5% yeast extract, 0.01% L-cysteine, 0.01% ammonium sulfate, 0.01% potassium phosphate dibasic, 0.002% magnesium sulfate anhydrous, 0.005% manganese sulfate monohydrate, and 0.04% potassium citrate for *L. fermentum* HEM0117; 4.0% glucose, 1.0% soy peptone, 1.5% yeast extract, 0.1% Tween 80, 0.3% potassium phosphate dibasic, 0.1% sodium acetate, 0.01% magnesium sulfate anhydrous, 0.01% manganese sulfate monohydrate, and 0.1% calcium chloride for *L. rhamnosus* HEM0042; and 2.0% glucose, 1.5% fructose, 0.07% whey protein, 0.5% fructo-oligosaccharide, 1.7% soy peptone, 2.0% yeast extract, 0.05% L-cysteine, 0.05% mono-sodium glutamate, 0.05% ascorbic acid, 0.1% Tween 80, 0.4% potassium phosphate dibasic, 0.1% sodium acetate, 0.2% magnesium sulfate anhydrous, 0.005% manganese sulfate monohydrate, 0.01% ferrous sulfate, 0.05% calcium carbonate, and 0.1% potassium citrate for *L. paracasei* HEM0020. All media were steam-sterilized at 121 °C for 15 min just before inoculation. Cultivations were performed with a 3.5 L working volume in a 5.0 L bench-top fermenter for all strains and pH was maintained at pH 5.5 with 2.0% (*v*/*v*) ammonia water for cultures of *L. paracasei* HEM0020 and *L. fermentum* HEM0117, and at pH 5.8 for cultures of *L. rhamnosus* HEM0042. Cultivations were performed for 12–16 h, depending on the growth rate of each strain, and then bacteria were harvested.

### 4.3. Preparation of Powdered Samples

Cell pellets were harvested by centrifugation (6387× *g*, Model No. Supra R22A; Hanil Scientific Inc., Seoul, Republic of Korea) for 20 min, and then mixed with the following (%, *w*/*w* of cell pellet): 5.0–10.0% skim milk, 5.0–10.0% trehalose, 1.0–5.0% sorbitol, 0.1–1.0% lysine, 0.1–1.0% gellan gum, 5.0–15.0% sodium phosphate dibasic, and 5.0–15.0% potassium phosphate monobasic for *L. rhamnosus* HEM0042, *L. fermentum* HEM0117, and *L. paracasei* HEM0020. The mixture was immediately frozen at −70 °C for 24 h.

The frozen samples were dried using a freeze dryer (Model No.: LP-10; IlshinBiobase, Seoul, Republic of Korea) by incrementally increasing the temperature from −50 °C to 25 °C over 72 h. Thereafter, the powders were harvested and crushed using a blender with a mesh size of 80. Finally, the powders were packed in aluminum pouches, which were stored at −20 °C prior to use.

### 4.4. Preparation of Lysed, Heat-Killed, and Viable Probiotics

The number of viable bacteria in powdered samples was measured using the assay described in Section 4.5 on MRSA media at 37 °C for 48 h. Thereafter, the samples were diluted to 1.0–1.5 × 10^8^ colony-forming unit (CFU)/mL with phosphate-buffered saline (PBS). Suspensions of viable bacteria prepared using these procedures were heated twice at 100 °C for 15 min in an autoclave. No viable bacteria were detected in the suspensions after this heat treatment.

Lysates were prepared using identical suspensions. Briefly, 100 IU lysozyme (Sigma-Aldrich, Saint Louis, MO, USA; CAS No., 12650-88-3; Model No., L6876-5G) was added to 10 mL of the suspension and incubated at 30 °C for 30 min. Sonication was performed for a total of 6 min (three times for 2 min) with an amplitude of 22 µm, a pulse of 10 s, and a pause of 20 s. Samples were maintained in an ice bath during sonication. Thereafter, cell disruption was confirmed using a microscope (1000×; Axioplan, Carl Zeiss, Jena, Germany). Particles were removed by syringe filtration using a filter with a pore size of 0.22 µm (Minisart; Satorius Stedim Biotech GmbH, Göttingen, Germany) before samples were used.

### 4.5. Viability Assay

The CFU assay was performed to investigate the viability of the probiotics in the powders. MRS agar was used to measure the number of viable bacteria in the powders after freeze drying. In total, 1 g of powder was suspended in 9 mL of autoclaved PBS containing 0.1% Tween 80, and then serially diluted by 10^8^, 10^9^, and 10^10^. Thereafter, 0.2 mL of the dilutes was spread on the surfaces of MRS agar plates and incubated in anoxic conditions at 37 °C for 48 h. Finally, colonies were counted (cfu/g of powder).

### 4.6. Preparation of Mouse Spermatozoa

ICR mice were purchased from Orient Bio Co., Ltd. (Seoul, Republic of Korea). All animal experiments, breeding, and care procedures were performed following the regulations of the Institutional Animal Care and Use Committee (IACUC) of CHA University. IACUC approval (approval number IACUC 230027) was obtained before initiation of the study. Male mice (*n* = 100, 8 weeks old) were utilized. Sperm were collected from the caudal epididymis and vas deferens regions.

### 4.7. Motility and Viability Analysis of Probiotic-Treated Sperm

Motility of spermatozoa treated with probiotics for 30 and 60 min was analyzed. The density of spermatozoa was adjusted to 20–50 × 10^6^/mL with HTF medium (MR-070-D; Sigma-Aldrich, MO, USA) supplemented with 5 mg/mL bovine serum albumin (A0281-1G; Sigma-Aldrich, MO, USA). Total sperm was analyzed by microscopy. Probiotic-treated sperm were transferred dropwise to 100 µL medium on a glass bottom dish. Dishes were observed with an inverted microscope (ECLIPSE Ti2–E; Nikon, Tokyo, Japan) at 6000× magnification. Six areas in each sample consisting of 1000 spermatozoa per field were analyzed for sperm motility and head morphology by elliptic Fourier analysis. These were analyzed by blind analysis by a different observer.

For vital staining, we used a vital staining solution consisting of 2% (*w*/*v*) Fast Green FCF (F7258-25G; Sigma-Aldrich, MO, USA) and 0.8% (*w*/*v*) Eosin B (E4382; Sigma-Aldrich, MO, USA) dissolved in human tubal fluid (HTF) media. An equal volume of semen was mixed with an equal volume of vital reagent (Eosin + Fast Green) and incubated for 30 s. The mixture was then smeared on a slide glass and dried on a slide warmer. The live (white color) to dead (red color) sperm ratio of each sample was then determined by light microscopy (ECLIPSE-E100; Nikon, Japan).

### 4.8. Western Blotting

Sperm that had been treated with probiotics for 30 min were immediately harvested, centrifuged, and washed with PBS which lacked Ca^2+^ and Mg^2+^. All samples were maintained at −80 °C until Western blot analyses. Proteins were extracted with PRO-PREP™ protein lysis buffer (17081; iNtRon Biotechnology, Seongnam, Republic of Korea). Samples were boiled with 4× Laemmli sample buffer (161-0747; Bio-Rad, Hercules, CA, USA) for 5 min, and 20 μL of each boiled sample was loaded into the well of an 8% sodium dodecyl sulfate polyacrylamide gel. Gels were electrophoresed at 80 V for 20 min, followed by 100 V for 30 min. Proteins were transferred to a nitrocellulose membrane (Bio-Rad) at 350 mA for 2 h. The membrane was incubated for 1 h with blocking buffer (Tri-buffer saline containing 0.1% Tween-20: TBST) containing 5% bovine serum albumin, and then with a primary antibody solution overnight at 4 °C. The primary antibodies were rabbit anti-mTOR (2983T; Cell Signaling, Boston, MA, USA), rabbit anti-phospho-mTOR (5536S; Cell Signaling), mouse anti-phospho-tyrosine (SC-7020; Santa Cruz Biotechnology, Dallas, TX, USA), mouse anti-AMPKα-1 (Aho1332; Thermo Fisher, Waltham, MA, USA), rabbit anti-phospho-AMPKα-1 (2531S; Cell Signaling), rabbit anti-AKT (MA5-14916; Thermo Fisher), rabbit anti-SIRT1 (2028S; Cell Signaling), and mouse anti-β-actin (MA5-15739; Thermo Fisher). Next, the membrane was washed with TBST and incubated with horseradish peroxidase-conjugated anti-mouse immunoglobulin G (Bio-Rad) for 1 h at room temperature. Immunoreactive bands were detected using an enhanced chemiluminescence detection reagent (Clarity™ Western blot substrate, 1705061; Bio-Rad). Images of the bands and their intensities were analyzed using a chemiluminescence imaging system (WSE–6100 LuminoGraph I; ATTO, Tokyo, Japan). The intensity of each band was analyzed with ImageSaver version 6 (ATTO, Tokyo, Japan) and normalized to that of the β-actin band. The experiment was repeated three times with different samples and then numerical analyses of the normalized intensity of each target band were performed.

### 4.9. Statistical Analysis

All data are expressed as means ± standard error of the mean (SEM) of triplicate measurements. Statistical analyses were performed using a one-way analysis of variance with the significance level set at *p* < 0.05. Significant differences are indicated by asterisks in the figures.

## 5. Conclusions

We found that probiotics enhance spermatozoa motility via mitochondrial function-related factors like AMPK and SIRT1. Administration of probiotics is a potential new strategy to improve sperm motility and treat male infertility.

## Data Availability

The original contributions presented in this study are included in the article. Further inquiries can be directed to the corresponding author(s).

## Supplementary Materials

The supporting information can be downloaded at: https://www.mdpi.com/article/10.3390/ijms252313220/s1.
