# Supplementary material for: The Probiotics Lacticaseibacillus paracasei, Lacticaseibacillus rhamnosus, and Limosilactobacillus fermentum Enhance Spermatozoa Motility Through Mitochondrial Function-Related Factors
Source: Int J Mol Sci. 2024 Dec 9;25(23):13220. doi: 10.3390/ijms252313220 (PMC11642019; doi:10.3390/ijms252313220)
Supplement: Supplementary file 1 [file ijms-25-13220-s001.zip › ijms-3146067-supplementary.pdf]

Supplement table

Table S1. Spermatozoa motility upon treatment with probiotics

	**30 min (%)**	**60 min (%)**
**Control**	17.45 (±3.77)	8.62 (±0.90)
L. paracasei **(10^2 dilution)**	36.63 (±6.17)	24.41 (±1.79)
L. fermentum **(10^3 dilution)**	33.76 (±4.08)	16.15 (±0.55)
L. rhamnosus **(10^4 dilution)**	32.83 (±4.25)	16.89 (±2.70)

Table S2. Spermatozoa viability upon treatment with probiotics

	**30 min (%)**	**60 min (%)**
**Control**	21.67 (±3.76)	12.17 (±4.09)
L. paracasei **(10^2 dilution)**	30.89 (±7.43)	26.83 (±8.28)
L. fermentum **(10^3 dilution)**	35.04 (±8.91)	26.11 (±9.8)
L. rhamnosus **(10^4 dilution)**	37.11 (±14.66)	24.17 (±9.40)
